# What passive euthanasia is

**DOI:** 10.1186/s12910-020-00481-7

**Published:** 2020-05-14

**Authors:** Iain Brassington

**Affiliations:** CSEP/ Law, University of Manchester, Oxford Road, Manchester, M13 9PL UK

**Keywords:** Euthanasia, Passive euthanasia, James Rachels, Intent, Withholding treatment, End-of-life care

## Abstract

**Background:**

Euthanasia can be thought of as being either active or passive; but the precise definition of “passive euthanasia” is not always clear. Though all passive euthanasia involves the withholding of life-sustaining treatment, there would appear to be some disagreement about whether all such withholding should be seen as passive euthanasia.

**Main text:**

At the core of the disagreement is the question of the importance of an intention to bring about death: must one intend to bring about the death of the patient in order for withholding treatment to count as passive euthanasia, as some sources would indicate, or does withholding in which death is merely foreseen belong to that category? We may expect that this unclarity would be important in medical practice, in law, and in policy. The idea that withholding life-sustaining treatment is passive euthanasia is traced to James Rachels’s arguments, which lend themselves to the claim that passive euthanasia does not require intention to end life. Yet the argument here is that Rachels’s arguments are flawed, and we have good reasons to think that intention is important in understanding the moral nature of actions. As such, we should reject any understanding of passive euthanasia that does not pay attention to intent.

**Short conclusion:**

James Rachels’s work on active and passive euthanasia has been immensely influential; but this is an influence that we ought to resist.

## Background

Euthanasia is a controversial and emotive topic, but normative debate about it resembles trench warfare. People who are in favour of liberal laws tend to stay in favour of liberal laws, but rarely persuade those who aren’t to change their minds; and the same applies, mutatis mutandis, in respect of people who are in favour of tight restriction or criminalisation. There is little argumentative innovation; what work there is tends to be – as Søren Holm has indicated – a matter of fortifying established positions [1]. It is rather as if the soldiers in opposing trenches, accepting that there is no territorial breakthrough to be made, have resigned themselves to half-heartedly throwing gravel at each other in the hope that they might dent their way to victory. In this context, another paper on euthanasia may be seen as attritional at best. It is not hard to see why.

The intention of this essay is not to make any normative claim about assisted dying. Rather, it is built around the idea that a significant shift in the debate is likely to require conceptual clarity, and that a *fruitful* shift certainly will; and that there is significant unclarity surrounding a number of aspects of euthanasia that impedes any such shift.

I shall focus in particular on an unclarity in the term “passive euthanasia”. The first part of my argument is that there are at least two very different understandings of the term in use. This means that participants in debates are often talking past each other. This is bad enough; but the second part of the argument contends that one of the candidate definitions of “passive euthanasia” is deeply misleading. This generates (and prevents the simple solution, or dissolution, of) significant problems for patients and medical staff who have to make decisions in practice, and for the lawyers who may have to give an opinion on those decisions. The argument echoes many of the arguments mounted by Garrard and Wilkinson in 2005 [2], in which they take aim at James Rachels’s highly influential claims from 1975 [3] about active and passive euthanasia.^1^ This is more than pleonasm, because – as we shall see – though the position articulated by Rachels is flawed, the influence of his paper persists, and it is undesirable that mistaken claims should be influential.

## Main body

### Defining euthanasia

I shall take as my point of departure Heather Draper’s definition of euthanasia, which is something of a touchstone. For her, death by euthanasia

must be defined as death that results from the intention of one person to kill another person, using the most gentle and easy means possible, that is solely motivated by the best interests of the person who dies [5].^2^

It is reasonable to infer from this that any instance in which means other than the gentlest and easiest possible are selected to bring about death is not an instance of euthanasia proper. This is because if death by euthanasia is motivated by the best interests of the person who dies – which may be taken to be a necessary though not sufficient part of euthanasia’s definition – it would always be in their better interests for the gentler available option to be used, the best being by definition that than which there is no better. Therefore, intentional killing that does not use the gentlest and easiest possible means cannot be euthanasia. At best, it is botched euthanasia; but it may not even be that, since we would presumably want to distinguish between instances in which the gentlest means are not selected, and those in which they are but in which their administration (for some reason) goes wrong.

Draper does not stipulate that death should be requested; this means that, under her definition, non-voluntary euthanasia is possible. On the other hand, it seems reasonable to assume that a positive desire to remain alive is trumps when making judgements about best interests, and that the phrase “involuntary euthanasia” is consequently an oxymoron. Neither does Draper make any stipulation about the identity of the person who does the killing, or about the relationship between the killer and the killed. This seems correct. Who the agents are might make a difference to the permissibility of a given instance of euthanasia – that is a possibility that merits investigation, but in another paper – and it might be the sort of thing lawmakers ought to consider; but it is not central to whether a putative mercy-killing is euthanasia by definition.

I am now in a position to offer this as a simplified definition of euthanasia:

#### Definition 1

*Euthanasia is the intentional ending of one person’s life by another, motivated solely by the best interest of the person who dies.*

Note the distinction between intent and motivation. Motivation refers to a general desire to secure the best interests of the patient, intent to the particular actions performed in the service of that motivation. This will prove important later.

A potential difficulty concerning motivation and intent is that either may be unclear or impure. Agents can, and frequently do, have mixed motivations for this or that action; further, agents may be psychologically opaque even to themselves, and unable as a result to say precisely why they did something.^3^ It follows from this that it may be difficult truly to assess motive and intent, and correspondingly difficult to say with certainty whether a given instance of ending a person’s life is, in the real world, truly an instance of euthanasia. That said, the fact that there may be obscurity on this matter does not mean that there always will be. Sometimes, and possibly often, assessments of motive and intent will be straightforward; and for the sake of the conceptual clarification that is the concern of this essay, possible psychological opacity matters little. We will only stand a chance of assessing whether an action fits the definition of euthanasia if we can get that definition straight in the first place; and getting a definition straight does not depend on how likely it is that any putative instance of euthanasia really is properly so called. In what follows, we can assume that the motives and intents attributed to actors are nothing more or less than they appear to be.

We may append to Definition 1 a distinction between *active* and *passive* euthanasia. The former would describe an instance in which Alice brings about Bob’s death, intentionally and motivated solely by Bob’s best interest, by performing some procedure on Bob.^4^ The latter would describe an instance in which Alice brings about Bob’s death, intentionally and motivated solely by Bob’s best interest, by withholding something necessary for his life to continue. A deliberate overdose of barbiturates might count as active euthanasia; removing a ventilator, or deciding never to ventilate to begin with, might count as passive euthanasia. (For the sake of fluency, “withholding” is used throughout this essay to cover both situations in which a treatment regime is not begun, and situations in which a treatment regime once begun is removed: withdrawal should be thought of as withholding treatment *from this time forward*.) Thus two more definitions can be offered, each of which is narrower than Definition 1:

#### Definition 2:

*Active euthanasia is the intentional ending of one person’s life by another, motivated solely by the best interest of the person who dies, through the deliberate administration of a life-ending substance or procedure.*

#### Definition 3:

*Passive euthanasia is the intentional ending of one person’s life by another, motivated solely by the best interest of the person who dies, through the deliberate withholding of a life-preserving substance or procedure.*

In essence, the difference between active and passive euthanasia on this account is the difference between adding something that otherwise wouldn’t be there, and removing something that otherwise could be.

However, Definition 3 is not reflected in a great deal of the medical, medical ethical, and medicolegal literature. A few examples might be offered, in chronological order. Moreland wrote in a 1988 paper that “[p]assive euthanasia occurs when a patient is *allowed to die* by withholding or withdrawing life-sustaining treatment” (emphasis mine) [6]. Similarly, when writing about voluntary active euthanasia in 1992, Dan Brock took pains to distinguish his topic from “withholding or withdrawing life-sustaining treatment, which some commentators characterize as ‘passive euthanasia’” [7].^5^ A letter to the *BMJ* from 2007 insists that passive euthanasia “whereby medical treatment is stopped and nature is allowed to take its course” is available and sometimes endorsed by the courts in the UK [8]. For Lo Ping-cheung,

‘Active euthanasia’ refers to terminating life via action, i.e., causing death by commission [ … ]. ‘Passive euthanasia’ refers to terminating life via inaction, i.e., causing death by omission [9].

On an archived web-page, undated but copyrighted to 2014, the BBC states that

[p]assive euthanasia occurs when the patient dies because the medical professionals either don't do something necessary to keep the patient alive, or when they stop doing something that is keeping the patient alive [10].

Alanazi and Alanzi have written that “[a]n example of passive euthanasia is simply *letting a patient die* without providing necessary treatment to save or prolong that patient’s life” (emphasis mine) [11]. In a 2016 paper, Varelius refers to “withholding and withdrawing life-supporting treatment from non-competent physically ill or injured patients” as a kind of passive euthanasia [12]. Potter treats “passive euthanasia” as referring straightforwardly to the withdrawal of treatment at the end of life [13]; Nair-Collins asserts that “passive euthanasia means to allow death to occur [and] is more commonly simply termed withholding or withdrawing life-sustaining treatment” [14].

Doubtless one could produce more examples with a little searching. Though there is a few cosmetic differences between the understandings listed in the last paragraph, those understandings are fundamentally alike in allowing for the idea that passive euthanasia is simply the withholding or withdrawal of life-sustaining treatment; the requirement that death be intended is absent. As such, the definition upon which they build is something like this:

#### Definition 3*

*Passive euthanasia is the ending of one person’s life by another, motivated solely by the best interest of the person who dies, through the deliberate withholding of a life-preserving substance or life-preserving procedure.*

Under Definition 3, though all instances of passive euthanasia are instances of withholding treatment, and though withholding treatment *may* count as passive euthanasia, not all instances of withholding will – only those in which treatment is withheld with the intention of ending life.^6^ Under Definition 3*, no appeal is made to any such intention: all instances of passive euthanasia are instances of withholding treatment, and, provided the motivation is the best interests of the one who dies, all instances of withholding treatment are instances of passive euthanasia. Alice need not intend Bob’s death. One possible implication of Definition 3* is that acceding to a refusal of treatment, or to an advance directive refusing treatment, could count as a form of passive euthanasia. Though such accession would not necessarily satisfy the medical best interests of the patient, we ought to keep in mind that medical best interests do not exhaust best interests *sensu lato*, which would include a patient’s desires. Being motivated to withdraw treatment because it has been competently refused may therefore plausibly count as being motivated by the best interest of the person who dies. As such, quite a lot of fairly routine treatment decisions may turn out to be forms of passive euthanasia if we accept Definition 3* that would not count as passive euthanasia according to Definition 3.^7^

(Draper and Slowther appear to endorse Definition 3* when they say that

([a] ctive euthanasia is said to have occurred when the person intending the death took some positive action to ensure this outcome – such as administering a lethal dose of medication.

(Passive euthanasia occurs when an action that could have prevented death is not taken (e.g. antibiotics are not given) or an intervention that is keeping death at bay is withdrawn (e.g. artificial ventilation) [15].

(However, since intention is mentioned on the previous page as part of the broader definition of euthanasia, it is not unreasonable to take it as implicit in the definition of passive euthanasia, which brings it back into line with Definition 3.)

Definitions 3 and 3* cannot both be correct – an intention to end life either is or isn’t part of the definition of passive euthanasia – and if nothing else, intellectual tidiness requires that we work out which (if either) is. However, there is more riding on this than tidiness. An instance of withholding treatment may or may not count as passive euthanasia depending on whether one cleaves to Definition 3 or Definition 3*; and in jurisdictions in which passive euthanasia is illegal, that will mean that determining the legality of withholding will depend on the definition chosen: if all withholding is passive euthanasia and all euthanasia is illegal, then all withholding will be illegal too. Ensuring that the term “passive euthanasia” is correctly understood will therefore clearly be a matter of great importance to the patients, medical staff, and lawyers involved in such decisions. Indeed, determining whether passive euthanasia is legal at all may depend on the definition that one chooses. In this light, and though any more thoroughgoing analysis belongs elsewhere, one might note that when Markandey Katju, the judge who delivered the opinion in the Indian case of *Shanbaug* [16],^8^ determined that passive euthanasia is legal in India, he did so on an understanding of the term that appeared to bounce between Definition 3 and Definition 3*, but which relied more heavily on the latter. Withholding treatment was deemed lawful, and was understood as passive euthanasia; and this led to the determination that passive euthanasia was lawful. But if law is built on terms that are not clearly defined or consistently used, that would seem to store up problems for the future. And, of course, further problems are stored up if any chosen definition is unconvincing in its own right. Clarity is important.

### Withholding and withdrawal of treatment understood as euthanasia

Can this confusion be untangled? The most straightforward way would be to show that one of the candidate definitions of passive euthanasia as mistaken; and the position put and defended throughout the rest of this essay is that it is Definition 3* that we should abandon. It follows from this that any ethical or legal opinion that builds on it should be seen as shaky at best. To help make the case, it is worth taking a quick look at the genealogy of Definition 3*.

When Alanazi and Alanzi [11] call withholding treatment passive euthanasia, they cite Malm’s paper on killing and letting die [18], and appeal to arguments made by James Rachels. Malm, in turn, also cites Rachels. Google Scholar says that Rachels’s 1975 paper on active and passive euthanasia has been cited over a thousand times to date; and via innumerable lectures to innumerable students, its influence will have gone further than might be suggested by the number of published citations. It is in this paper that, without actually defining active and passive euthanasia, Rachels puts forward the claim that

[t]he distinction between active and passive euthanasia is thought to be crucial for medical ethics. The idea is that it is permissible, at least in some cases, to withhold treatment and allow a patient to die, but it is never permissible to take direct action to kill a patient [3].

Now, none of the definitions of euthanasia offered above makes any claim about permissibility – and normative claims do not have an obvious place in attempts to define any term. However, from the structure of the passage from which this quotation is taken, it is fairly clear that Rachels wants us to link killing (of which people tend to disapprove) with active euthanasia, and letting die (in respect of which people tend to be more flexible in their attitudes) with passive. It is not unreasonable to see in this the genome of Definition 3*. And a couple of pages later, he asks and answers a rhetorical question:

Why do so many people think there is an important moral difference between active and passive euthanasia? One reason is that they believe the more general proposition that *killing someone is morally worse than letting someone die* [3].

For sure, it might be that Rachels is simply indicating that “many people” draw a close link between passive euthanasia and letting die; we do not have to think that he endorses it based on this quotation. In itself, this does not tell us much about the nature of that link. Indeed, an association between passive euthanasia and letting die, on the grounds that the former is a form of the latter, is perfectly within the scope of Definition 3, and so those who think that Definition 3 is correct and 3* incorrect should have no difficulty here. (The difference between Definition 3 and Definition 3* on this front is that adherents to the former will want to deny that the truth of the claim that anyone who commits passive euthanasia is letting the patient die implies the truth of the claim that anyone who lets a patient die is committing passive euthanasia.) On this reading, all Rachels is saying is that if and when letting die is permissible, then so is killing, and that this means that if passive euthanasia is permissible, then so is active.

However, the tone of the passage allows for the inference that there is more than an association between the two – that there is something more like identity. It is perhaps notable that, while the bulk of his essay concerns the difference between killing and letting die, its title is simply “Active and Passive Euthanasia”. Rachels has drawn the dots and given us a pen with which to join them. More explicitly, in *The End of Life*, he writes that

[b]y ‘active euthanasia’ we mean taking some positive action *designed to kill* the patient; for example, giving a lethal injection of potassium chloride. ‘Passive euthanasia’, on the other hand, *means simply* refraining from doing anything to keep the patient alive [4].

The emphasis in this quotation is mine. Rachels links active euthanasia with a particular design that he does not link to passive. In his account of passive euthanasia, two things are absent: treatment, and a requirement that the end of life be intended.

A parenthetical point worth making here is that Rachels looks for support for his claim about what many people think about active and passive euthanasia to a statement from the American Medical Association. Thus, he says,

[t]his doctrine [of the moral difference between active and passive euthanasia] seems to be accepted by most doctors, and in 1973 it was endorsed in the first policy statement ever issued by the American Medical Association. That statement said, in its entirety:

The intentional termination of the life of one human being by another – mercy killing – is contrary to that for which the medical profession stands and is contrary to the policy of the American Medical Association.

The cessation of the employment of extraordinary means to prolong the life of the body when there is irrefutable evidence that biological death is imminent is the decision of the patient and/or his immediate family. The advice and judgment of the physician should be freely available to the patient and/or his immediate family.

In subsequent statements the AMA refined its policy, but the central idea [ … ] has remained [3].

Note, though, that this statement is only in accordance with a claim about the difference between active and passive euthanasia, and the moral difference between them, if one has already decided that all letting die is passive euthanasia. Absent that idea, then the AMA’s position is simply that euthanasia is ruled out, and allowing to die is not. If we look at the latest iteration of the AMA’s policy, this is clearer: Opinion 5.8 sets out its opposition to the permissibility of euthanasia, which it defines as “the administration of a lethal agent by another person to a patient for the purpose of relieving the patient’s intolerable and incurable suffering”. There is no mention of withdrawal or withholding treatment. Where withholding and withdrawal *are* mentioned, it is in Opinion 5.3, at which point there is no mention of passive euthanasia [19]. In other words, the AMA appears to lean away from Definition 3*. That said, Rachels does complain about the second paragraph of the AMA’s statement, “for what is the cessation of treatment [ … ] if it is not ‘the intentional termination of the life of one human being by another’?” [3] – but, given his appeal to the AMA’s authority to get his argument going in the first place, this is a touch ironic. (I shall have more to say about whether one could cease life-sustaining treatment without intending death in a little while.)

Be that as it may: it is plausible to hold that Rachels’s paper, which perhaps owes its influence to having appeared first in the *New England Journal of Medicine*, has played a crucially important role in the widespread acceptance of Definition 3*.^9^ What remains to be seen is whether Rachels (or the position with which he has become associated) is correct. If he is not, Definition 3* – and the legal opinion that relies on it – is in trouble. And there are compelling reasons to think that he is not.

### Rachels on killing and letting die

Much of the donkey-work in Rachels’s argument is done by a thought-experiment concerning Smith and Jones. In a nutshell, Smith drowns his young cousin in order to secure an inheritance; Jones notices his young cousin drowning, and, mindful of the inheritance, is delighted and attempts no rescue [3, 4]. Rachels’s argument is that

1. Smith killed and Jones let die;
2. It is hard to see how there is a moral difference between Smith’s and Jones’s cases;
3. Therefore it is hard to see how there is a moral difference between killing and letting die.
4. Active euthanasia is killing; passive euthanasia is letting die.
5. Therefore there it is hard to see how there is a moral difference between active and passive euthanasia.

If this is an accurate reconstruction, it should be clear that Rachels commits an important mistake in the move from (2) to (3), because he assumes that the reason for the moral similarity between Smith’s and Jones’s case will apply to all instances of killing and letting die; and this he does not argue. I shall have something more to say about that in a moment. But a further fallacy creeps in at (4), where passive euthanasia is taken to be not just a *kind* of letting die, but *identical* with it. To show that there is a fallacy, it would be useful to consider an example of an instance of letting die that we would not want to call passive euthanasia.

Let us, then, extend the thought experiment. Brown sees the drowning child, and, believing that she is suffering so severely that death would be better for her and that there is no more gentle way to bring it about, holds her under the water. Robinson sees the drowning child, and, believing that she is suffering so severely that death would be better for her and that there is no more gentle way to bring it about, does nothing to pull her out of the water. García sees the drowning child, and, believing simply that it would be impossible to get the child out of danger of lasting harm, does not intervene.^10^ In one sense, Brown is like Smith, since both kill intentionally, and Robinson and García are like Jones, since all let die. However, there are also important differences between these new cases and Smith and Jones’s. For sure, Brown and Robinson’s intents – that the child’s life should end – are similar to Smith and Jones’s; but García has a different intent from any. Further, our three new protagonists all have a different motivation from Smith and Jones, since there is no benefit from any will to be had. Yet if there are differences between Jones’s motivation and Robinson and García’s, and differences between García’s intent and all the others’, it would seem that not all instances of letting die are quite alike in anything but the most superficial sense; and if not all instances of letting die are alike, the way is open to asking whether one of them is better described as passive euthanasia than the others; and if one is better described as passive euthanasia than the others, then it cannot be true that having established that someone let someone else die will be sufficient to establish that they performed passive euthanasia.

It would be misplaced to consider Smith’s or Jones’ actions as instances of euthanasia – active or passive – since the child’s interests are plainly not served: the intention that the child’s life should end was not accompanied by the right sort of motivation. Neither satisfies Definition 1. Provided that there was no gentler way available of ending the child’s life, Brown committed active euthanasia, and Robinson passive: both were motivated by a belief about best interests, and both intended that the child’s life should end. What about García? Under Definition 3*, he also committed passive euthanasia. But, the intent being different, there is a certain gravity to the idea that his (in)action is qualitatively different from Robinson’s: García does not withhold intervention *so that* the child will die. As such, one might easily be drawn to say that applying the term “passive euthanasia” to what both Robinson and García do obscures an important distinction between letting die with the intention of ending life, and letting die generally. The moral importance of this distinction – which speaks to the move from (2) to (3) in the reconstructed argument above – can be acknowledged by anyone but the most implausibly naïve consequentialist. (Note, of course, that drawing a distinction between Robinson and García’s actions and insisting that it is morally salient does not have any implications about the permissibility of either, unless we think that unlike actions cannot be equally permissible.)

One possible response to this would be to split Definition 3* into two further subcategories: passive euthanasia in which death is the intended outcome, and passive euthanasia in which it isn’t. But those subcategories would match Definition 3’s differentiation between passive euthanasia and other instances of letting die. In other words, Definition 3* would turn out to be a version of Definition 3, but with added scope for confusion. It would be better to go directly to Definition 3.

This serves to rebut a possible argument along the lines that preferring Definition 3 to Definition 3* is simply stipulating that passive euthanasia is one thing rather than another, and thereby doing little more than gainsaying defenders of Definition 3*. Definition 3 is to be preferred to Definition 3* because, if we think that there is some kind of differentiation to be had between Robinson and García, Definition 3 promises a more precise and more efficient way of explaining it. This is why, incidentally, it will not do to defend Definition 3* on the grounds that meaning is use, that “withholding treatment” is what the term “passive euthanasia” has grown to mean in at least some contexts, and that that is that. Even if the term has shifted from Definition 3 to Definition 3*, we do not have to be indifferent about it if the shift makes the language that informs ethical and legal decisionmaking less precise. We can still argue in favour of precision.

This principle applies in clinical contexts. Imagine a variation on the Brown, Robinson, and García cases above. McDonald is a medical professional who, believing that his patient is suffering so severely that death would be better for her and that there is no more gentle way to bring it about, administers a fatal overdose in order to precipitate her death. Campbell is a medical professional who, believing that his patient is suffering so severely that death would be better for her and that there is no more gentle way to bring it about, withholds life-sustaining treatment in order to precipitate her death. Finally, Mukherjee is a medical professional who, believing that her patient will not benefit from it, withholds life-sustaining treatment. According to Definition 3*, all three would have committed a form of euthanasia, and Campbell and Mukherjee would have committed passive euthanasia. But it seems plain that Campbell’s action is intended to cause death; Mukherjee’s is simply one in which death is a side-effect. For sure, Mukherjee’s (or García’s) belief may be false, and her inaction may be blameable. But even if both Mukherjee and Campbell are blameable, and attract precisely the same amount of blame, that won’t show that they should both be described as having performed the same kind of action – of having performed passive euthanasia. It is preferable to treat Campbell’s action as passive euthanasia, and Mukherjee’s as simple withholding short of passive euthanasia.

### The importance of intent

Before pressing the case further, it is worth taking a moment to consider a possible line of objection that builds upon the idea that Rachels’ position is not being presented in the best possible light.

To recap: In the Smith and Jones case, the “no difference” principle rests on the idea that Smith and Jones both behave abominably, and that it does not matter in making that assessment that one acted to kill and the other let die. This Rachels takes to be evidence that there is not a moral difference between killing and letting die. If and when killing is wrong, then so is letting die; likewise, if and when it is not wrong, then neither is letting die. In the context of euthanasia, this line of thought is taken to serve as evidence that there is no moral difference between active and passive euthanasia: if one is permissible or wrong, then so is the other. As far as it goes, this argument seems fairly plausible: if our aim is that someone be dead, then the manner in which that aim is realised probably does not matter. Yet it may not go all that far; and this is because – *per* the argument in the last section – it blurs a distinction that we may want to draw between “kinds” of letting die: to wit, letting die when death is intended, and letting die when it is not. This is precisely the distinction upon which Definition 3 rides.

But Rachels thinks that the distinction between a death that one intends and a death that one does not intend is illusory. Though the Smith and Jones example may not show it all that clearly, the less widely-cited examples of Jack and Jill, and of Drs White and Black, which he introduces in his “More Impertinent Distinctions” essay [21], may help provide a better view of his position. If these examples provide us with a compelling reason to think that intent is irrelevant to how we should think about killing and letting die, the putative distinction between Definition 3 and Definition 3* will have to be abandoned – and, with it, any distinction between Robinson and García, or between Campbell and Mukherjee.

In the first example, Rachels introduces us to Jack and Jill. Each visits their grandmother in order to cheer her up; each expects that their visits will mean that their grandmother will become fonder of them, and bequeath them more in her will. The ostensible difference is that Jack predicts that a difference will be made to the bequest, but does not intend it; Jill visits *so that* a difference is made to the bequest. Yet, says Rachels, though we may think that Jack and Jill’s characters differ in morally important ways, we cannot deny that what they actually *do* – the physical business of turning up for a chat over tea and cake – is the same, and so must attract the same evaluation. The rightness, permissibility, or wrongness of a particular action does not depend on the intention behind it. And if that is so – if intention is unimportant and there is no distinction to be drawn between doing something *so that* something will come about, and doing it *even though* it will – it would seem to follow that there is no distinction between letting die and passive euthanasia. Definition 3* ought to be embraced.

Similarly, in the second example, White and Black are both confronted with the case of a severely ill infant whom it is impossible to save, and whose suffering may be increased by further treatment. White decides to withhold treatment, notwithstanding that death will be quicker than it could be; Black decides to withhold further treatment *so that* death will be quicker than it could be. Again, says Rachels, their (in)action is the same; again, there is no distinction to be drawn between killing and letting die. And this example is particularly pertinent to the question of passive euthanasia: we may think that either acted rightly, permissibly, or wrongly; but because they both did the same thing, what we say about one we have to say about the other. Again, there is no line between letting die and passive euthanasia. Again, if that is right, a proponent of Definition 3 would seem to be forcing a distinction where there is none.

How convincing is Rachels, and anyone who follows a similar argumentative strategy, here?

The answer to this question must be “Not very”. Through the White and Black example, Rachels certainly succeeds in showing that what Definition 3 would have us call letting die and passive euthanasia are equally permissible in some cases; and it might even be that they are equally permissible in all cases. From this, we can accept that he is right to say that what he calls the “traditional” position, that letting die is somehow less bad than killing, is mistaken. However, it does not follow from that that “passive euthanasia” is simply letting die, because while it would be true that *α* and *β* are equally permissible when all *α*s are *β*s, that does not entitle us to say that all *α*s are *β*s when they are equally permissible. Rachels’s argument, therefore, missteps here. More, my claim in this paper is precisely that, whatever the permissibility of passive euthanasia and letting die, they are different things; therefore we cannot reach any conclusions about the permissibility (and legality) of passive euthanasia simply by pointing out that letting die is or can be permissible (or legal).

So what are the flaws in Rachels’s position? Two stand out. The first is that his account of what Jack and Jill, and White and Black, do is somewhat threadbare. It is hard to make sense of an action, morally or metaphysically, without some account of intention. To stipulate that the physical movements of Jack, Jill, White and Black are the whole story in understanding what they did is to take a strange view of what an action is – it is something like insisting that there is nothing to a symphony except pressure waves in air. In one sense, this is true, of course; but if someone said that he would be out tonight because he was going to witness the Hallé Orchestra making pressure waves in air, we would wonder what, precisely, he had paid for. Music is not reducible to its bare physical characteristics; and neither is any other human endeavour. The movements that constitute actions are contextualised by projects, and understood by the part they are intended to play in those projects. By parity of reasoning, it seems that if someone tries to insist that killing and letting die are the same things in all respects just because the physical movements are the same, they have missed something important about the project upon with the person doing the movement has embarked.

The other flaw is that Rachels’s account seems not to be able to make sense of the distinction between success and failure. Imagine that Jill’s grandmother had sussed out exactly what was going on, and was therefore at the very best no better-disposed towards Jill than she had been before Jill visited. On Rachels’s account, there is no distinction to be had between what Jack and Jill did. But insofar as that Jack would have succeeded in his project while Jill would have failed in hers, this would seem to force us to say that neither is there any meaningful distinction between an action’s success and failure. Yet that looks absurd; we cannot claim to have understood an action if, as a matter of principle, we cannot say whether it did what it was supposed to do. Seen from the other side, it would become quite mysterious how it could be that Jack had succeeded in his project and Jill failed in hers, granted that they are supposed to have done the same thing. And so we turn out to have a reason to resist the idea that they had done exactly the same thing after all. The simplest way to make that distinction is to appeal to Jack and Jill’s intention. Or, to put it another way, intention has earned its place in understanding – and determining – what an action is.

To iterate: Rachels’s argument, we can allow, is perfectly sound when it comes to showing that different actions, or even different kinds of action, may be of exactly the same moral worth, and for corresponding reasons. White and Black both want the best for the child in their care, and this will be of central importance when deciding permissibility. But establishing moral congruence will not establish that we are talking about the same action, or the same kind of action, in each case. As such, there is a role for intention after all; and this means that Definition 3 is likely to be a better definition of passive euthanasia than Definition 3* even when the strengths of Rachels’s argument are taken into account, simply because of the role that intention plays.

### Foresight and intent

The argument so far rests on the supposition that Mukherjee does not intend the patient’s death, and that it is on this basis that she did not commit euthanasia. But, on the assumption that she is a tolerably competent medic, she must have foreseen that death as the likely outcome of withholding life-sustaining treatment; and that withholding was itself intentional. We might be tempted to think that we cannot really divorce the death from the intent, and that the death was therefore intentional after all. (As we have seen, this is a position that Rachels seems to endorse.) This might mean that any non-negligent instance of letting die really is passive euthanasia.

Yet this objection rests on an unwarranted conflation of the foreseen outcome of an intentional action, and its intended outcome. That the two are not the same can be easily demonstrated. Suppose I am considering whether or not to have another glass of wine. I have a reason not to: as a martyr to hangovers, I think I will be fine in the morning if I abstain, but that a further drink will make me feel dreadful. On the other hand, the wine is good, I am enjoying the conversation that accompanies it, and so on. All things considered, I decide to help myself to another glass. Now, it is certainly true that the ensuing headache would be a foreseen consequence of an intentional action, and I could only really say that it was my own fault. But it would be quite a stretch to say on the basis of that that I intended to have a hangover. As I sip my wine, I hope that this will be the exception to the norm, and that I will, for a change, escape any particularly deleterious consequences. This is sufficient to show the difference between foresight and intent: it would be very strange to say that I might intend something the non-appearance of which would be a relief.^11^

Yet if I can intentionally drink another glass of wine without intending to get the hangover that nevertheless I expect, much the same would appear to be the case for Mukherjee. There is no reason at all to suppose that in intentionally withholding of life-sustaining treatment she intends that the patient should die. Mukherjee could even acknowledge that that would be the overwhelmingly likely outcome; it would still be coherent for her to hope for the patient’s unexpected survival, just as I could hope to avoid a hangover that I think more or less certain. This would not be the case for McDonald or Campbell. This is a point that was articulated well by Garrard and Wilkinson in their 2005 paper. Their understanding of passive euthanasia requires, in accordance with Definition 3, that “the main purpose (or one of the main purposes) of withdrawing or withholding is to bring about (or hasten) the patient’s death” [2; slightly altered]. And, they argue,

whether the healthcare professional’s intention is to bring about death can be established by using the following counterfactual test: if the patient does not die, has the health carer succeeded in his or her aim? In the case of passive euthanasia, the answer will be ‘no’ because the health carer was aiming at the patient’s death. But in the case of withdrawing or withholding treatment because it would be burdensome or harmful, then the health carer can have succeeded in this aim – to protect the patient from a particular burden or harm – even if the patient pulls through without treatment [2].

That the patient’s survival would have thwarted Campbell’s project and not Mukherjee’s is vitally important, and shows that there is a significant difference between the moral natures of their respective projects. That there is this difference in their moral natures will tell us nothing very much about whether either is permissible – maybe Campbell should be praised, or maybe Mukherjee was negligent; but it provides us with much firmer ground on which to base any subsequent normative claim or evaluation. What matters is that the reasons behind the assessment cannot be facsimiles of each other.

### Acts of omission

If the above is correct, it shows that there is a thoroughgoing distinction to be drawn between Definition 3 and Definition 3* of passive euthanasia, and that Definition 3 is preferable. This is for three reasons. First, it tracks the important distinction between withholding treatment *so that* the patient dies, and withholding treatment *even though* the patient dies. Second, it makes discussion of passive euthanasia clearer, since it relieves us of the burden of having to come up with a further way to talk of the so-that/even-though distinction. Third, the relationship of Definition 3 to Definition 1 mirrors neatly the relationship of Definition 2 to Definition 1. The difference between Definition 2 and Definition 3 is simply one of whether positive action is present or absent; intention is conserved. As noted above, Definitions 2 and 3 each therefore narrow the scope of Definition 1. Definition 3*, by contrast, is somewhat more complicated, inasmuch as that it narrows the scope of Definition 1 it in one respect (by stipulating that it refers only to instances in which treatment is withheld), but widens it in another (by removing any stipulation about intent). This consideration may not be definitive; but it is also not unreasonable to think that clear thinking about passive euthanasia is easier when the relationships between definitions are as simple as possible.

But the presence or absence of positive action invites a further take on the question of the nature of passive euthanasia.

Consider this passage from Rachels’s “Active and Passive Euthanasia”:

It is not exactly correct to say that in passive euthanasia the doctor does nothing, for he *does do one thing* that is very important: he lets the patient die. “Letting someone die” is certainly different, in some respects, from other types of action – mainly in that it is a kind of action that one may perform by way of not performing certain other actions [3].

Consider also this, from his essay “Killing and Letting Die”, originally in the *Encyclopedia of Ethics*:

So what is the difference between causing and allowing? What real difference is marked by those words? The most obvious ways of attempting to draw the distinction won’t work. For example, suppose we say it is the difference between action and inaction – when we cause an outcome, we do something, but when we merely allow it to happen, we passively stand by and do nothing. This won’t work because, *when we allow something to happen, we do perform at least one act: the act of allowing it to happen*. The problem is that the distinction between doing something and not doing something is relative to the specification of what is or is not done – if I allow someone to die, I do not save him, but I do let him die [22].

Rachels’s position here must be that allowing a death is a kind of action. But, that being the case, we might wonder what exactly is passive about it. It looks rather as though Rachels is hinting that there might be no such thing as passive euthanasia, and that putatively passive euthanasia is active in at least some sense after all, because even an omission is a kind of action.

On this theme, it is worth noting that, in an article about the *Shanbaug* case, Rohini Shukla has suggested that withholding treatment is one thing, and withdrawing it quite another, such that only the former would count as passive euthanasia proper [23] – again, the thought here is that to withdraw treatment is a kind of action, and that withdrawal is therefore a kind of active euthanasia. But even when talking about withholding, her claim makes use of one of the ways that some people have tried to make sense of passive euthanasia, which is to advert to the phrase “act of omission”. Thus

[w]ithholding life support implies that crucial medical intervention is restrained, for example, not performing a kidney transplant when it is necessary for the patient’s survival. This would involve acts of omission on the part of the doctor [23].

Shortly after this, she claims that “in passive euthanasia the doctor only passively commits acts of omission” [23]. It is hard to say what “passively committing” an act is; but what matters is the deployment of the idea of an “act of omission”. It would seem to be very like what Rachels was talking about in his insistence that not to treat is a kind of action in its own right.

Shukla is not the only one to use the phrase. For example, Sawyer et al write that

[a]ctive euthanasia is a positive act of commission, such as a lethal injection; passive euthanasia implies an act of omission, such as the withholding or withdrawal of treatment [24].

This is echoed by DiBaise, whose formulation of passive euthanasia is similar:

Euthanasia can be active, as when someone intentionally chooses to kill a person by an act of commission, or it can be passive, as when someone brings about the death of a person by an act of omission [25].

But what are we to make of the phrase “act of omission”, or the thought that withholding treatment is, despite appearances, a kind of action?

A moment’s thought shows that we should probably be nonplussed. Rachels’s position, on examination, seems tautologous at best, “letting” and “allowing” being much the same in standard English. Talking about “acts of omission” might break the tautology, but both its meaning and the motivation for appealing to it are obscure. It is possible that the phrase reflects a belief that only actions can stand moral scrutiny, and that we must treat everything as an action if we are to say anything morally interesting about it. But that would fly in the face of any insistence that there is no moral distinction between active and passive euthanasia *qua* killing and letting die, and there is no reason at all to suppose that all things that we would want to praise or blame must be actions anyway. Negligence can be established in ethics simply by showing that an agent did not do what she ought to have done; we don’t have to show that something *was* done as a part of that.

The metaphysical legitimacy of the idea of an “act of omission” would seem to rest on a number of tacit, but ultimately unconvincing, assumptions. One of these we might call *hyperpositivism*. Hyperpositivism is characterised by the insistence that every decision is a (positive) decision to *φ*, rather than a (negative) decision not to *ψ*. Thus, a negative decision to withhold treatment is actually a disguised positive decision – either to not-treat (as though that is a verb in its own right), or to do something else. Yet it should be clear that hyperpositivism is fallacious. In the first place, it seems demanding: having decided that a decision is a decision to *φ*, rather than not to *ψ*, we must scrabble around to find something for *φ* to be. It is more parsimonious not to give ourselves this job. More fundamentally, it is simply false that a decision not to *ψ* is actually a decision to *φ*. For example: suppose I had planned to go to the cinema this evening, but that my mood changed, and I find myself no longer so inclined. Although we might *say* that I had decided to stay at home instead, this is a figure of speech: we should not conclude that staying at home was a positive decision. Rather, staying at home is the default. It’s what one does if one doesn’t go out. One doesn’t have to choose to do it, because it’s not really doing anything at all. It’s what one “does” – and the word “does” should perhaps be used only with some hesitation – if one is suddenly overtaken by a wave of indifference to everything; and inertia is neither an action nor conducive thereto. If it were, it wouldn’t be inertia. Equally, we would not want to say that *φ* represents the action of not-going-to-the-cinema, which I had chosen over going to the cinema, because not-going-to-the-cinema is not an action. It is an omission pure and simple, and there is no need to try to force it to be anything else.

Another tacit assumption, which is a *sequela* to hyperpositivism, is that there is always a clear thing for *φ* to be. This, too, is a little hard to swallow, for there is an indefinitely long list of things that *φ* could be, and it might well be that we can only identify which is the correct candidate retrospectively. This leaves us wide open to accusations of having fallen victim to the *post hocergo propter hoc* fallacy. Suppose that, having decided against going to the cinema, I watch *A Space Odyssey* on DVD for the two-thousand-and-first time, or bake, or draft a rather pedantic essay for an academic journal. Conceivably, someone might say, “Ah-ha! Brasso *chose* to draft an essay instead of going out!” as though this is a discovery about my state of mind when I chose not to go to the cinema. But putting to one side the possibility that I simply sit and stare blankly into the middle distance until overcome by sleep, I have to do *something* to fill the gap between now and bedtime: I have time free, which I subsequently fill. But finding oneself *φ*-ing is not the same as choosing to *φ*, and choosing to *φ* to fill the gap left by not *ψ*-ing is not the same as choosing *φ* over *ψ*. (I take it as a given that we would want to resist any line of argument that would force us to assert that I might have *chosen* to stare blankly into the middle distance simply for want of anything better to assert.) Working the other way, it might well be the case that someone did not *ψ* because she was too busy *φ*-ing; but in cases like this, it is the omission of *ψ* that bothers us, not the commission of *φ*, and we can make sense of claims about the former – claims about whether it was negligent, for example – without having to impute claims about the latter.

Now, a fairly obvious response to all this is that it misses the point that there is an important distinction to be made between withholding treatment based on a belief about a shorter life being better for the patient than a longer one, and simply not providing it because, say, one forgot, or was otherwise engaged. “Euthanasia” could only ever be used in situations in which an agent *decides* to withhold treatment; and inasmuch as that a positive choice is required, this is where the act in an act of omission is to be found. Hence the phrase “act of omission” should not be all that perplexing after all. And, as far as it goes, this line of thought is fair enough; but it leaves untouched two important points. The first is that it does not address the distinction between deliberately withdrawing treatment *so that* life will be ended, and deliberately withdrawing treatment *even though* life will be ended – that is, the distinction between Definitions 3 and 3*. The second is that it also blurs the distinction between Definition 2 and Definition 3, since the agent would be positively “doing” something in all circumstances. For sure, some people might think that this is all to the good – that the distinction between active and passive euthanasia is a distinction without a difference anyway. Such a position is tenable. But in that case, we would be justified in saying that one should not talk about passive euthanasia at all, since there turns out to be no such thing (and arguments about the difference between Definition 3 and Definition 3* would be revealed to be barely even scholastic). Yet inasmuch as that people *do* talk about passive euthanasia, and talk about it in certain ways, the position outlined here stands. Whether or not they should is a question for another paper.

If this is correct, it suggests that we can talk about omissions perfectly straightforwardly without having to think that all omissions are kinds of action. We don’t have to talk about what Campbell or Mukherjee *did* to make moral sense of events; it’s enough to say that they did not treat, and then to concern ourselves with the presence or absence of an intention to end a life. We might want to talk about what they did instead; but we do not have to. Talk about “acts of omission” simply adds layers of nouns for the sake of propping up a dogma about agency that we have no obvious reason to maintain to begin with. It is for the best if we steer clear of the whole wobbly edifice. Acts are acts; omissions are omissions; and there is no particular reason to suppose that a medical decision to withhold treatment, *qua* omission, has to be an “act of omission”. Since parsimony is a virtue, we shouldn’t.

### The importance of clear definitions

Rachels’s paper (and the position that it reflects) is correct insofar as that it claims that, when ending someone’s life is wrong, it does not matter whether it is by killing or letting die. And because there need be no normative distinction to be drawn between killing and letting die *per se*, neither need there be one to be drawn between active and passive euthanasia, since they are species of killing and letting die. However, by giving insufficient attention to motive and intent, Rachels obscured the important differences that *do* obtain between some instances of letting die, and, on that basis, that can be discerned between passive euthanasia and other kinds of letting die. This obscurity has persisted throughout a great deal of work, both within and without the academy.

Why does any of this matter? Why is this not simply a pettifogging paper about the use of a small phrase? There is a handful of reasons.

In the first place, even in the absence of any normative claims about how we should evaluate killing and letting die, what should be clear is that one cannot skip straight from a claim about the permissibility of one to the permissibility of the other. It might be that killing a patient by withholding treatment is permissible in at least some cases, and maybe in many; but this cannot be just because letting die often is. Passive euthanasia is a kind of letting die, but it is not reducible to letting die. This matters because it provides us with a lens through which we can examine statutes and judicial opinions and the way they are interpreted, and the dicta of judges in different regimes. The bare fact that a phrase like “passive euthanasia” may get used differently in different times and places is not of all that much importance in itself; but how legal systems or commentators *understand* the term, and the plausibility of each understanding, is. If there is ambiguity about something like the meaning of “passive euthanasia” as it is used within a legal regime, we may expect that the quality of legal decisionmaking, or policymaking, would suffer; as Chan and Tse note when considering passive euthanasia in a Hong Kong context,

it would not help public discussion if the term ‘passive euthanasia’ is used indiscriminately without a clear definition, especially when non-controversial cases of forgoing [life-sustaining treatment] are referred to as ‘euthanasia.’ [26]

Indeed, if ambiguity leads to confusion about the legality of passive euthanasia, it may leave medical staff and those in associated roles in danger of violating the law unwittingly. For example, if they are told that passive euthanasia is legal, and interpret this to mean that they may intentionally end someone’s life, when the authority that assured them of its legality meant simply that it was legal only to withhold unhelpful treatment, they may commit a serious, life-ending, blunder. (Relevant here is the claim by Cipriani and Fiorino that English law allows for passive euthanasia based on the assumption that passive euthanasia is nothing but the withdrawal of treatment [27]. That, and how, a medical professional who takes such a statement at face value may end up in serious trouble hardly needs spelling out.) Seen from a certain perspective, things might be almost as bad for the patient in a regime where euthanasia is illegal, and who is kept alive by means of burdensome medical treatment administered by medical staff labouring under the impression that withdrawing treatment is euthanasia and therefore forbidden.

A remedy to problems like this might well depend on settling on a secure and compelling definition. So which definition should we choose?

I have argued in this paper that there is a significant difference between what I called Definition 3 and Definition 3*, and that there are good reasons to prefer the former. Further, it is reasonable to think that this should be reflected in law; for even if law built on shaky definitional foundations is law for all that, it is nevertheless reasonable to prefer that the law at least aspire to stability. A law that is blind to a distinction between *φ* and *ψ* for any given *φ* and *ψ* is a less acute law than it might be, and potentially a flawed law.

## Conclusion

The core claim of this essay is that there are two possible and incompatible ways to understand the term “passive euthanasia”. On one understanding, presented here as Definition 3, an intent to end life is required; on the other, presented as Definition 3*, the action that ends life may be intended, but the end of life itself need not be. Despite the prevalence of Definition 3*, the argument has sought to defend the position that Definition 3 should be preferred. Withdrawing life-sustaining treatment when death is not the intended outcome – and it may not be^12^ – is not euthanasia at all, passive or otherwise. A sub-theme of the argument has been that the prominence – even if not necessarily the provenance – of Definition 3* can be traced to James Rachels’s work, and to its reception in the literature since; idea here has been that if Rachels’s argument is mistaken, then claims that rely on it or that have the same structure must also be rejected. In this light, it is important to note that even if Rachels himself would have fought shy of the claim that all instances of withholding treatment are instances of passive euthanasia, there is enough in the work that he produced to sustain Definition 3*; and even the most charitable reading of Rachels would still force us to admit that his understanding of the term “passive euthanasia” is unclear and his use of it is confusing.

It would be false to claim to be the first to take on the task of clarifying the meaning of “passive euthanasia”. The position outlined here is very much in line with that adopted by Garrard and Wilkinson in 2005 [2]. Indeed, as long ago as 1979, Bonnie Steinbock was arguing against Rachels’s claims about the AMA [29]. And yet, as we have seen, versions of Definition 3* recur and hold sway in the academic literature and beyond. For this reason, it is worth making the argument again, if only for the sake of fortifying the trenches in which the wider normative battle is fought. Passive euthanasia should not be identified with withholding of life-sustaining treatment. Only some instances of non-treatment, instances in which the intention is to end life, are instances of passive euthanasia.

## Data Availability

N/A

## Footnotes

1 As well as calling his 1975 NEJM paper “Active and Passive Euthanasia”, Rachels would appear to have used this same title for a chapter in his *The End of Life* [4]. Happily, and with only one exception, everything by Rachels to which I refer in this essay is – at least currently – available for free from www.jamesrachels.org.

2 p. 176

3 I am grateful to Simona Giordano for bringing this point to my attention.

4 The editors have asked me to make clear that Alice and Bob, and all the other characters in the thought-experiments in this essay, are fictional. An exception to that rule applies when I choose to place myself as a character in a thought-experiment: I believe that I am real. I trust that the context will make all clear.

5 We cannot infer that Brock aligns himself with those who characterise withholding or withdrawal as passive euthanasia; he is simply pointing out that such people exist.

6 This is clear in Draper’s definition: “Withholding of therapy is not always passive euthanasia” [5].

7 I am grateful to Margot Brazier and to Dieter Birnbacher for pressing me for clarification on this point. By instinct, I would want to resist the idea that it would be euthanasia to withdraw treatment at the patient’s request; but that is not sufficient to show that Definition 3* is mistaken. That said, as we shall see, I think that Definition 3* *is* mistaken.

8 In brief, this case considered the legality of withdrawing treatment from a woman who had been in a prolonged vegetative state, and who was expected never to recover. That euthanasia was mentioned at all in the *ratio* is indicative of certain presumptions about the relationship between (passive) euthanasia and withdrawal of treatment. See my “How Not to Talk About Passive Euthanasia”, currently in press with the *Indian Journal of Medical Ethics*, for a fuller analysis of the case [17].

9 1975 also saw the publication of a paper called “Should the Patient be Allowed to Die?” by Richard Nicholson [20], on page 6 of which passive euthanasia is defined as “a failure to use therapies that would prolong life in a patient with a terminal illness”; but it is fair to say that this paper has been much less influential.

10 It seems to me that there is not a straightforward parallel with García in Rachels’s original scenario.

11 The arguments here would obviously apply to debates about the doctrine of double effect, too.

12 cf. the similar point made by Joel Gamble et al: “Causing death could be in patients’ and physicians’ intentional landscapes in [withdrawal of care], but it need not be.” [28]
